# The application of explainable artificial intelligence (XAI) in electronic health record research: A scoping review

**DOI:** 10.1177/20552076241272657

**Published:** 2024-10-30

**Authors:** Jessica Caterson, Alexandra Lewin, Elizabeth Williamson

**Affiliations:** 1 4615 Imperial College London, London, UK; 2 4906 London School of Hygiene and Tropical Medicine, Bloomsbury, UK

**Keywords:** Artificial intelligence, digital, digital health, eHealth, electronic, health, health informatics, machine learning, systematic reviews

## Abstract

Machine Learning (ML) and Deep Learning (DL) models show potential in surpassing traditional methods including generalised linear models for healthcare predictions, particularly with large, complex datasets. However, low interpretability hinders practical implementation. To address this, Explainable Artificial Intelligence (XAI) methods are proposed, but a comprehensive evaluation of their effectiveness is currently limited. The aim of this scoping review is to critically appraise the application of XAI methods in ML/DL models using Electronic Health Record (EHR) data. In accordance with PRISMA scoping review guidelines, the study searched PUBMED and OVID/MEDLINE (including EMBASE) for publications related to tabular EHR data that employed ML/DL models with XAI. Out of 3220 identified publications, 76 were included. The selected publications published between February 2017 and June 2023, demonstrated an exponential increase over time. Extreme Gradient Boosting and Random Forest models were the most frequently used ML/DL methods, with 51 and 50 publications, respectively. Among XAI methods, Shapley Additive Explanations (SHAP) was predominant in 63 out of 76 publications, followed by partial dependence plots (PDPs) in 11 publications, and Locally Interpretable Model-Agnostic Explanations (LIME) in 8 publications. Despite the growing adoption of XAI methods, their applications varied widely and lacked critical evaluation. This review identifies the increasing use of XAI in tabular EHR research and highlights a deficiency in the reporting of methods and a lack of critical appraisal of validity and robustness. The study emphasises the need for further evaluation of XAI methods and underscores the importance of cautious implementation and interpretation in healthcare settings.

## Introduction

### The ‘Black Box’ problem

Artificial intelligence (AI) in healthcare is anticipated to transform the sector.^1–7^ One example is machine learning (ML) and deep learning (DL) models for predictive analytics using tabular electronic health record (EHR) data.^8^ EHR data are a wealthy resource, providing extensive information which can be applied for many beneficial applications including predicting 30-day mortality,^9^ identifying risk groups for future diseases,^10,11^ and estimating hospital length of stay.^12^

However, a limitation of ML/DL applications in this context is that how or why the predictions are made from a set of covariable, or feature, values is generally unclear.^1,13–17^ Such explanations are necessary in order to justify, control, improve and discover new information from any model.^14^ Justification is important to ensure patient and clinician trust in application of these models in health,^15,18,19^ and to comply with ethical and legal expectations. Any AI model implemented in health settings will need to gain trust from the patients it is used on, and the clinicians and organisations that use it. Failure to do so may risk the under-utilisation of AI in areas where it can be of significant benefit.^20^ From an ethical perspective, humans should be treated as such, not as objects, and thus ‘human in the loop’ approaches are preferrable.^21^ Legally, there are an increasing number of rules and regulations which set expectations around the explainability of AI.^21^ For example, the General Data Protection Regulation (GDPR) stipulates the right to an explanation and to contest any decision relating to automated processing and a person's health.^22,23^ GDPR also highlights the importance of informed consent in the context of automated decision making,^23^ a sentiment echoed in other legal documents such as the European Chart of Fundament Rights.^24^ Control and improving models are essential to ensure any predictive model is not biased, and protect the model from adversarial attacks.^14,19,25–27^ If predictions can be explained, then the underlying mechanisms for disease could be discovered – however, extra care must be taken in separating an association from a causal pathway.^18,28–30^

This lack of interpretability of complex ML/DL methods is referred to as the ‘black box problem’.^1,13^ This contrasts to methods such as generalised linear models (GLMs), decision trees (DTs) and rule-based methods, which are ‘intrinsically interpretable’. That is they generate easily interpretable outputs which are intuitively explainable to humans.^18^

### Explainable artificial intelligence (XAI)

A proposed solution for interpretation of ‘black box’ models is explainable artificial intelligence (XAI).^14,19^ XAI encompasses a range of interpretation methods which can be applied to black box models to generate interpretable explanations for their predictions. Approaches include ‘model-specific’ methods limited to one or some ML/DL models, and ‘model-agnostic’ applicable to any ML/DL model.^31^ Methods may also be ‘intrinsic’, i.e., they arise from the model itself (just as coefficient weights are produced from GLMs, and tree visualisations from decision trees), or ‘post-hoc’, requiring further analysis for the explanation.^31^ There are also ‘transparent models’, which are designed to retain the high-performing predictive capabilities of ML/DL, but have the interpretability of simpler models.^15,18,19^ They include generalised additive models (GAMs)^32^ and Bayesian Rule Lists (BRLs).^33,34^ However, their predictive performance is regarded as inferior to standard ML/DL methods, and thus they are not seen as a viable alternative at present.^15^

Among XAI methods, model-agnostic post-hoc approaches are preferrable, preserving flexibility for model selection and interpretation comparison.^31^ Post-hoc methods also do not require the underlying model to be altered, and so can be developed without access to the original model training data. These approaches can be further sub-divided according to the nature of the explanation provided into: (1) individual explanations (2) global explanations and (3) feature/outcome relationships.

#### Individual explanations

Individual explanations provide information for why a specific prediction was made e.g., why a particular patient with certain characteristics is predicted to developed breast cancer within the next 5 years. This is advantageous when explaining black box model predictions, because many of the modelling methods employ multi-dimensional, complex, non-linear explanations which are difficult to generalise across all individuals.^31,35^ By focussing explanations on one individual of interest, individualised XAI methods offer an explanation that is locally representative.^35^

XAI methods for individual explanations include Local Individualised Model-Agnostic Explanations (LIME),^35^ Shapley Values,^36^ SHapley Additive exPlanations (SHAP)^37^ and ‘Counterfactual’ explanations.^31^

The LIME method creates a locally representative explanation by building a linear model around an individual of interested and surrounding data points (e.g., the patient of interest and patients with similar characteristics) weighted inversely by their distance from the point of interest.^35^ The prediction is explained by coefficient weights derived from the linear model for each feature. The weight of the data points around the individual of interest is defined heuristically.^35^ Thus, explanations are vulnerable to changes in the weighting used.

Shapley values are derived from economic game theory, where they calculate the ‘pay-out’ to each contributor working in a coalition to generate profit.^36^ In XAI, the contributors are the features in the model, and the ‘profit’ is the predicted outcome. Shapley values represent the contribution of each feature to the final prediction; the larger the contribution, the larger the Shapley value ‘pay-out’.

SHAP values are derived from Shapley values. However, they are constructed such that the sum of the SHAP values for all of the features for a single prediction is equal to the final prediction. This is an example of an additive attributive model.^37^ In doing so, SHAP values can be aggregated across predictions to give global and feature/outcome relationship explanations. Calculation of SHAP values is computationally intensive, thus in practice, a user-defined sample of data is usually taken, and the LIME method is used to calculate the SHAP values.^31,37^

Counterfactual explanations utilise an interpretation approach commonly used by humans where something is explained by describing what would need to happen for the prediction to change.^31^ In the context of predictive models, this involves illustrating how the predicted outcome changes given changes in input features/variables.^38^ This is favourable to allow non-technical audiences to conceptualise unknown modelling processes by associating change of input with output.^39^ However, they do not necessarily equate to causality, which is a clear risk of their application in healthcare settings.

#### Global explanations

Global explanations provide an overview of how features contribute to predicted outcomes.^31^ Examples include global surrogate models and feature importance.

Global surrogate models train an intrinsically interpretable model on the features from the original data and predicted outcome from the black box model, to generate an intrinsically interpretable model which – hopefully – closely replicates the predictions from the more complex black box model.^31^ The explanation from the intrinsically interpretable model can then explain how the predictions are generated from the original black box model. It is also important to ensure that the interpretable model is a reasonable proxy for the black box model. Molnar (2023) suggests this can be done by calculating the *R*^2^ between the predictions from the black box model and those from the surrogate model.^31^

Feature Importance describes multiple methods which rank features used to train a model in order of some metric of ‘importance’. The earliest feature importance was designed for Random Forests.^40^ This method retrains the model with each feature's values consecutively shuffled, and compares the difference in ‘impurity’ (i.e., the lack of discrimination between the true and predicted outcome) between each model and the original model. The greater the difference in impurity, the more important that feature. Later, this method could apply to any model type by comparing prediction error.^41^ Feature importance is a useful aid for feature selection, but is less reliable for model interpretation as it depends on the scale of the feature's values. In addition, it may be influenced by unrealistic permuted data values.^31^ Features may also be regarded as less important if correlated with each other.^31^

Contrastingly, SHAP feature importance takes the absolute SHAP value for each feature for all individuals in the data set and averages the value.^37^ It then ranks features in importance of average absolute SHAP value. This is a truly ‘post-hoc’ and ‘model-agnostic’ approach and, rather than describing error, quantifies overall contributions of each feature to predicted outcomes.

#### Feature/outcome relationships

Feature/Outcome relationships specify how changing a feature value changes the prediction, including non-linear and multi-dimensional associations.^31^ Relationship explanations can also show how different features interact with each other by colouring plots according to an additional feature's value. Relationship explanations are often displayed graphically as a feature value against prediction, and thus are typically limited to showing 1-2 features at a time due to the limits of human dimensional perception.^42^ Examples of methods include partial dependence plots (PDPs), individual expectation curves (ICE) and accumulated local effects (ALE) plots.

Partial dependence plots show how a prediction changes over a range of feature values across their marginal distribution. That is, overall values of other features in the dataset. The relationship is calculated using the partial dependence function, which for a given feature, takes each individual's data and calculates the predicted outcome for that feature value with the remaining values fixed.^42–44^ The final relationship is an average of how the prediction changes over all individuals. A risk of using these plots is over-interpreting relationships for specific feature values which are very rare or improbable in the dataset. This can be addressed by showing density plots, which show how the feature values are distributed. ICE plots are very similar, except they display each individual as a separate curve on the plot.^45^ This helps to show heterogeneity in changes in prediction between individuals.

ALE plots use the conditional instead of marginal distribution of the feature being changed.^31^ This avoids improbable or impossible combinations of values,^31^ for example a person of height 2 m, who weighs 40 kg, or a person aged 50 who has smoked for 45 years.

It is also possible to display SHAP dependence plots.^37^ These plots display the relationship of feature value to its SHAP value across a dataset.

### XAI in healthcare research

Research using tabular EHR data are increasingly adopting XAI methods. A scoping review conducted by Payrovnaziri et al. (2020) from 2009 to 2019 identified only 42 publications using XAI, with only 5 employing post-hoc and model-agnostic methods.^46^ LIME appeared in two publications,^47,48^ and a global surrogate model in one.^49^ Unique methods included a probability calculation for feature importance by Eck et al.,^50^ and automated/manual rule pruning by Luo et al.^51^ A later survey conducted by Di Martino and Delmastro (2023) up to 2021 reported 71 publications using post-hoc model-agnostic XAI in tabular and time-series EHR research, with 90% published from 2020 onwards.^52^ SHAP was the most frequently used interpretation method in this survey.^52^

## Study aims/objectives

The aim of this scoping review is to provide an up-to-date overview into the use of post-hoc model-agnostic interpretation methods for ML and DL in EHR tabular research. It also aims to characterise and critically appraise the use of frequently applied methods in practice.

## Methods

The scoping review was conducted in accordance with PRISMA guidelines for scoping reviews.^53^ Table 1 provides the inclusion and exclusion criteria. Two major scientific literature databases, PUBMED and OVID/MEDLINE (including EMBASE) were searched for publications.

**Table 1.** Inclusion and exclusion criteria applied for the scoping review literature search.

Inclusion criteria	Exclusion criteria
Publication relating to the use of interpretation methods (XAI) to explain machine learning models in a healthcare setting. Post-hoc model-agnostic XAI used Tabular electronic healthcare data research Original articles, reviews, letters Text available in English	Outside scope (no XAI, intrinsic or model-specific XAI only) Wrong Data Type (Related to genetics, imaging, natural language processing, or sensor data) Full Text Unavailable

XAI = explainable AI.

**Figure 1.**
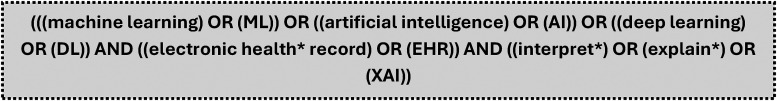
Search terms for PubMed and OVID/MEDLINE (including EMBASE) database.

The review comprised three phases: an initial broad search, title and abstract review, and full-text review. The initial search was conducted on 1st May 2023 and an updated search was completed on 27th June 2023, using the search terms provided in Figure 1.

All publications including original articles, reviews, and letters were included in the initial search, published any year, and available in English. The purpose of starting with a broad search field was to capture the wide range of terminology and publication types across computer science and medical science research. The online systematic review platform Rayyan was used to carry out publication screening.^54^ In the two subsequent phases of screening, title and abstract, and full text, the exclusion and inclusion criteria in Table 2 were applied. Publications were included if they used post-hoc model-agnostic XAI to explain ML models applied to tabular EHR data in healthcare. Decision to exclude articles was based on three criteria: out of scope (for example, no XAI, or only intrinsic or model-specific XAI e.g., methods specific to deep learning, neural networks), wrong data type (for example imaging, genetic data, or natural language processing), or if the full text was unavailable. A final search of grey literature from included publication citations and the Google search engine identified additional relevant articles.

**Table 2.** Frequency of metrics for evaluating and selecting the final ML model.

Metric	No. of publications (%)
AUROC	70 (92)
F-score	32 (42)
Accuracy	25 (33)
Recall (sensitivity)	47 (62)
Precision (PPV)	37 (49)
Specificity	25 (33)
NPV	10 (13)

AUROC = area under receiver operater curve, PPV = positive predictive value, NPV = negative predictive value.

Title, publication type, year of publication, author, journal, medical sub-specialty and study aim were recorded for each selected article. In addition, ML models used (including final selected model), metrics for model selection, and interpretation method(s). The nature of interpretation was analysed under three domains: (1) Global Feature Importance, (2) Feature/Outcome Relationships and (3) Individual Explanations. Data were analysed and summarised using R (v4.2.2).

## Results

**Figure 2.**
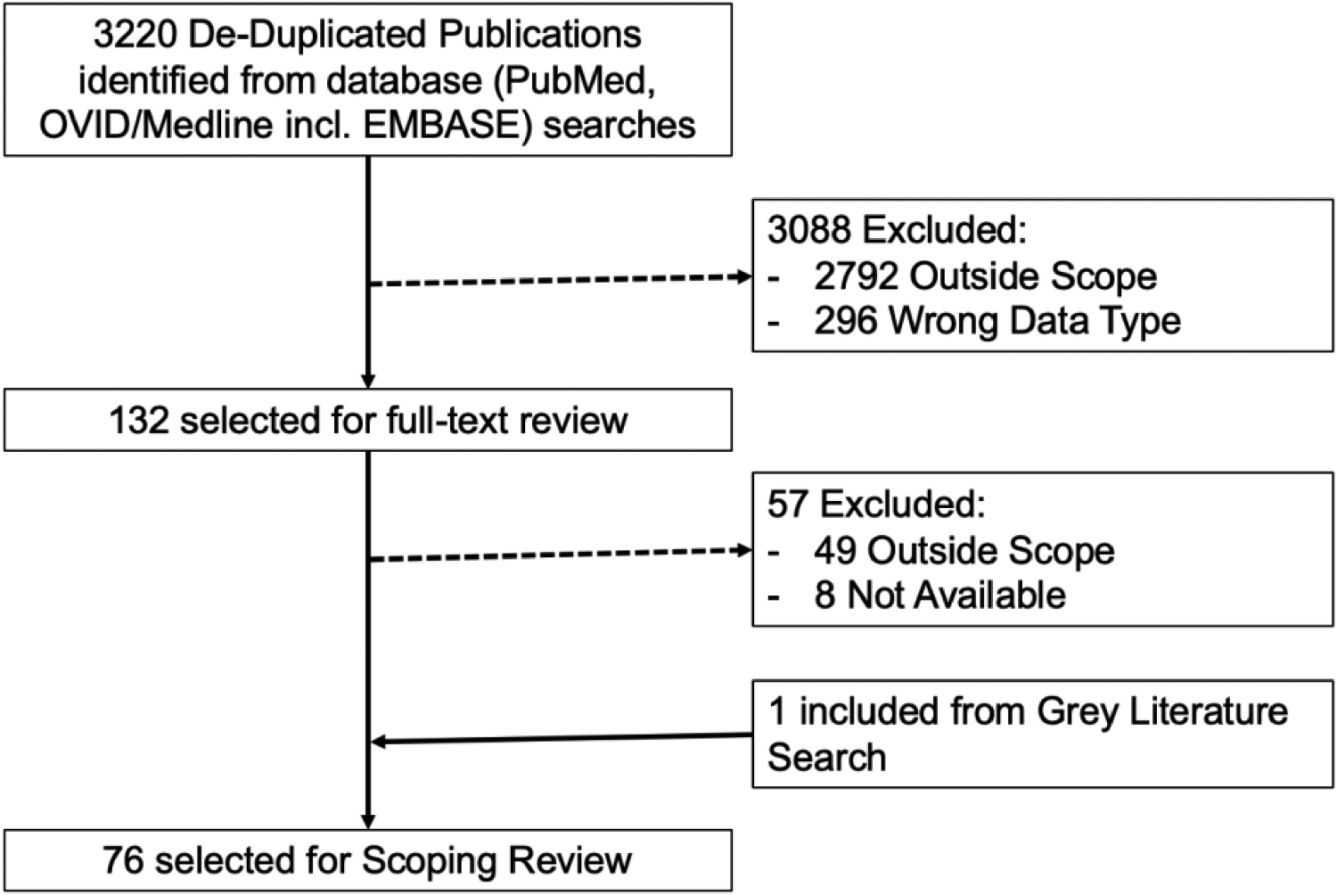
PRISMA scoping review flow chart.

After de-duplication, 3220 publications were identified from the search criteria. Of these, 132 were selected for full-text review. There were 2792 publications excluded for being outside the scope of the review and a further 296 related to clinical imaging or genetics. Of the 132 selected for full-text review, 75 were selected for final inclusion. An additional paper was included from the grey literature search. Figure 2 summarises the selection process.

### Publication characteristics

**Figure 3.**
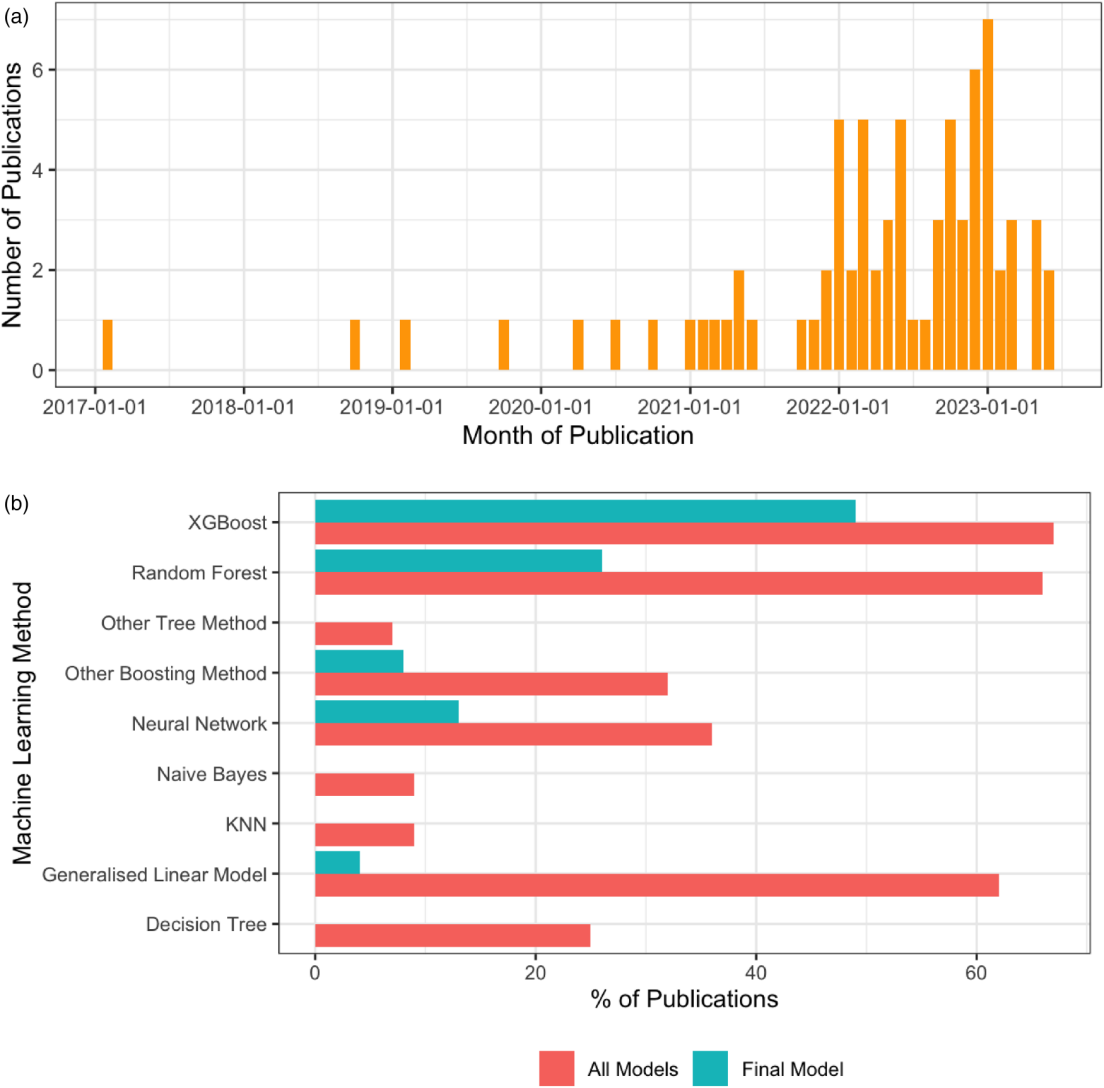
(a) Included publications over calendar time (b) Machine learning methods selected for testing and the final model as a % of publications.

All included publications were original peer-reviewed research articles. The articles were published between February 2017 to June 2023, increasing substantially in this time period (Figure 3a).

Identified studies aimed to improve predictive performance for classification of diseases, identifying complications and estimating length of stay using EHR data. Common domains were medicine (24 publications),^48,55–77^ COVID-19 (10 publications),^78–87^ Psychiatry (7 publications)^88–94^ and surgery (7 publications).^95–101^ Most publications emphasised interpretation methods for causal reasoning, especially for COVID-19, where knowledge of the disease was limited at the time of publications.^86,87^ Justification of the model's predictive performance was also a commonly cited motivation for using interpretation methods.^56,102–104^

### Machine learning models

Of the 76 publications, most trialled multiple ML/DL methods to get the best predictive performance. In the model development phase, EXtreme Gradient Boosting trees (XGB) were the most used in 67% (51/76) publications, followed by Random forest (RF) in 66% (50/76) of studies and Generalised linear models like logistic regression (LR) (Figure 3b). XGB remained the top choice for the final models, with RF also frequently selected (Figure 3b).

A range of performance metrics were evaluated for the selection of the final ML model (Table 2). Area under the receiver operating curve (AUROC) was the most frequently reported (70/76). F1-score, accuracy, recall (sensitivity), precision (i.e., positive predictive value, PPV) and specificity were also all assessed in over one-third of publications (Table 2).

**Table 3.** Methods for interpretation of machine learning and the number (percentage) of publications.

Method	Interpretation domain	No. of publications (%)
SHAP	Global, relationships, individual	63 (83)
PDP	Relationships	15 (20)
Feature importance	Global	11 (14)
LIME	Individual	8 (11)
Surrogate Model	Global	6 (8)
Shapley	Individual	3 (4)
ICE	Relationship	3 (4)
Rule-based	Global	1 (1)
Counterfactual	Individual	1 (1)
Other	–	7 (9)

### Interpretation method selection

Overall, 16 different interpretation methods were used (Table 3). SHAP was the most frequently used interpretation method, reported in 83% (63/76) of publications. LIME, PDP and surrogate models were used in 9 (12%), 8 (11%) and 7 (9%) of publications, respectively. Eleven publications included feature importance. Interpretation methods were used to evaluate only the final selected ML model(s) in all bar two publications, which used an interpretation method to compare how different models predicted outcomes.^55,84^

### Interpretation method application

#### Individual explanations

SHAP values were the most frequently used method for individual explanations, presented in 30 publications.^55,56,60,67,68,72–75,77,83,86,89,91,93,98–100,103–114^ Individual explanations using the SHAP method were displayed visually with force or waterfall plots.^111^ These plots show a series of arrows pointing right (positive SHAP value) or left (negative SHAP value), which together combine to reach the final predicted outcome.

LIME was used in eight publications.^48,55,73,84,97,102,115,116^ Sun et al. (2022) compared SHAP individual explanations with LIME, and inferred that their similar interpretations supported the ‘stability’ of the interpretations, which may be expected given SHAP values are calculated from the LIME method in practice.^73^ LIME was commonly used to contrast positive and negative predictions.^48,73,115,116^ Alternatively, Kibria et al. (2022) used LIME to compare outputs for one prediction across models to explain why different black box models had different predictive performances.^55^

Amongst all the publications that used SHAP or LIME, only one publication reported the heuristically defined hyper-parameters.^48^

Other, less frequently used individual explanations included counterfactual explanations. There was only one identified use of this method by Banerjee et al. (2021) to predict mortality in severe mental illness.^92^ However, rather than using this method causally as is the intended purpose, they reported a spurious result which they felt was indicative of bias in the dataset.

#### Global explanation methods

SHAP importance was the most frequently reported metric. In its simplest form, SHAP importance was represented as a bar plot.^60,63,67,69,70,73,75,77,80–82,86–90,98,99,101,104,105,110,113,117–125^ However, another commonly used figure was the ‘violin plot’, which shows the SHAP value for each instance in the data as a dot for each feature.^55–59,62,66,72,74,76,78,79,83,84,91,93–96,100,103,106,109,112,114,126–128^ Features are ordered from top to bottom from the most to least important. Each dot is coloured to represent if the feature value is high or low. In doing so, this plot not only shows the ranked order of importance of features, but how feature values may influence the SHAP value for each feature. Feature importance was used in 11 publications.^48,55,57,60,66,73,99,108,112,113,119^ It was typically used as simple summary of a model, followed by other explanations.^48,55,57,60,66,73,99,101,108,111–113,119,124^ In some cases, feature importance was compared with SHAP importance.^60,73,99,113,119^ The order of features according to permutation feature importance *versus* SHAP importance often differed, but was little commented on. This could have implications in later work, which may only select some features for further investigation based on one or other important metric.

Global surrogate models were used in six publications.^49,82,114,116,123,129^ This method was generally used to justify an underlying black box model, such as describing predictive pathways. Vyas et al. (2022), used a surrogate model to describe predictions for the diagnosis and severity of dementia.^116^ They explained their final (black box) model, a random forest, using a simple decision tree. From the decision tree, they identified groups at very high or low risk of developing dementia, and stated, for example, ‘if a person fails to identify all three animals [in a cognitive assessment questionnaire], they have a 95% chance of developing dementia’.^116^ Only one (Zhang et al. 2022^123^) publication quantified how well the surrogate model approximated the black box model. Zhang et al. (2022), reported *R*^2^ values of 0.68 and 0.57 for their support vector machine (SVM) and RF models, i.e., the proportion of variance explained between predictions from the black box model and surrogate model were 68% and 57%, respectively.^123^ These values implied there was a difference between the surrogate and underlying black box model, and may invalidate the surrogate model as a means to justify or provide an explanation for this prediction.

#### Feature/outcome relationships

Partial Dependence Plots were used in 15 publications.^49,61,66,71,73,79,100,101,108,109,111,121,125^ Relationships between features and outcomes were shown over a wide range of feature values, however few publications also showed the density of data at feature values from which the calculations were made.^37,79,109^

In two publications, individual conditional expectation (ICE) curves were also shown.^73,108^ In the paper by Sun et al. (2022), this was helpful in highlighting heterogeneity in feature/outcome relationships for some features, which can be hidden by showing only the average partial dependence alone.^73^

Sun et al. (2022) and Qiu et al. (2022) also presented SHAP dependence plots with PDPs to compare relationships.^73, 109^ The plots were generally similar in both studies, which may imply the techniques are robust. Interactions between features were shown using SHAP dependence plots in eight publications.^57,59,93,94,96,98,112,114^

## Discussion

This scoping review shows a significant rise in the interest and application of XAI methods within the last 5 years in EHR tabular research. In contrast to the 2020 review by Payrovnaziri,^46^ this review reports a 10-fold increase in post-hoc model-agnostic publications. This aligns with the study by Di Martino & Delmastro (2023) which reported a substantial rise in XAI publications up to 2021.^52^ Among the 76 identified publications, diverse method choices and motivations were evident. Some focussed on simple global feature importance metrics, whilst others only have individual explanations, and some gave comprehensive interpretations of global importance, feature/outcome relationships and individual examples.

The need for XAI was supported by the high rate of selection of black box models as the final model (e.g., Random Forest, XGBoost, Neural Networks), although there is likely a high selection bias towards these methods as simpler interpretable methods were less likely to need XAI in the first place. AUROC was overall the most favoured metric, whereas other metrics were seen in a third or less of publications. AUROC may have been favoured for its interpretation in the context of imbalanced datasets, which are commonplace in healthcare data. The range of other metrics selected may represent the diverse aims of the models developed which may favour a specific goal e.g., high sensitivity versus high specificity.

SHAP was the most popular method, not reported by Payrovnaziri et al. (2020),^46^ but by Di Martino & Delmastro (2023).^52^ This is likely because the SHAP method was introduced in 2017,^37^ and so only translated to healthcare research post-2019. Its widespread use suggests a perceived superior performance versus other methods, but authors seldom explicitly justify this choice.^52^ However, what is notable about SHAP compared with the other methods is that it offers interpretation approaches across all three core domains discussed. It also is more theoretically robust than other techniques.^31,37,52^ Thus, using the SHAP method enables authors to gain in-depth insight into their black box models, with a strong theoretical backing,^31^ without needing to use multiple methods.

The interpretation methods lack critical appraisal, with vulnerabilities such as susceptibility to adversarial attacks, as seen in instances like concealing ethnicity in the COMPAS dataset.^130^ Potential consequences, such as influencing predictions in healthcare decision-making, remain largely unexplored and unacknowledged in publications. Moreover, the dependence on heuristically defined hyperparameter values is evident,^35,37^ with limited reporting and understanding of vulnerability by authors.^48^ Evaluation of global surrogate models was also lacking, and in the one instance where it was performed, there was poor approximation between the black box and surrogate model.^123^ Evaluating similarity should be a requirement when using this method. Partial dependence plots were shown over wide feature value spaces, even though some feature values may be very rare or impossible.^31^ Greater awareness of the limitations of these interpretation methods is important to their appropriate application in the future.^52^

This scoping review is limited to tabular electronic health record research, and thus does not provide an overview of the application of interpretation methods to other healthcare domains such as genetics, imaging, NLP and time-series analysis. XAI in these domains differs from tabular EHR research, due to the nature of the data involved,^52^ and thus the applicability of XAI methods. This review also does not report model-specific methods, methods limited to subdomains of AI such as deep neural networks, or transparent models, and thus may not include other widely used methods limited to these groups. However, these methods are arguably inferior to model-agnostic approaches, given they limit the flexibility in initial model selection.^14,15,31,52^

## Conclusion

Overall, this scoping review reinforces the recent surge in the application of post-hoc interpretation methods for addressing the black box problem in health care. There is significant heterogeneity in the choice and application of these methods. There is also a lack of evaluation of XAI methodologies. Further research is required to properly assess these methods for their intended purpose and ensure that they are appropriate, robust and stand up to the demands of explanations expected within healthcare.

## Supplemental Material

sj-pdf-1-dhj-10.1177_20552076241272657 - Supplemental material for The application of explainable artificial intelligence (XAI) in electronic health record research: A scoping review Supplemental material, sj-pdf-1-dhj-10.1177_20552076241272657 for The application of explainable artificial intelligence (XAI) in electronic health record research: A scoping review by Jessica Caterson, Alexandra Lewin and Elizabeth Williamson in DIGITAL HEALTH
